# Separation of Plasmacytoid Dendritic Cells from B-Cell-Biased Lymphoid Progenitor (BLP) and Pre-Pro B Cells Using PDCA-1

**DOI:** 10.1371/journal.pone.0078408

**Published:** 2013-10-30

**Authors:** Kay L. Medina, Sarah N. Tangen, Lauren M. Seaburg, Puspa Thapa, Kimberly A. Gwin, Virginia Smith Shapiro

**Affiliations:** Department of Immunology, Mayo Clinic, Rochester, Minnesota, United States of America; Oklahoma Medical Research Foundation, United States of America

## Abstract

B-cell-biased lymphoid progenitors (BLPs) and Pre-pro B cells lie at a critical juncture between B cell specification and commitment. However, both of these populations are heterogenous, which hampers investigation into the molecular changes that occur as lymphoid progenitors commit to the B cell lineage. Here, we demonstrate that there are PDCA-1^+^Siglec H^+^ plasmacytoid dendritic cells (pDCs) that co-purify with BLPs and Pre-pro B cells, which express little or no CD11c or Ly6C. Removal of PDCA-1^+^ pDCs separates B cell progenitors that express high levels of a Rag1-GFP reporter from Rag1-GFP^low/neg^ pDCs within the BLP and Pre-pro B populations. Analysis of Flt3-ligand knockout and IL-7Rα knockout mice revealed that there is a block in B cell development at the all-lymphoid progenitor (ALP) stage, as the majority of cells within the BLP or Pre-pro B gates were PDCA-1^+^ pDCs. Thus, removal of PDCA-1^+^ pDCs is critical for analysis of BLP and Pre-pro B cell populations. Analysis of B cell potential within the B220^+^CD19^−^ fraction demonstrated that AA4.1^+^Ly6D^+^PDCA-1^−^ Pre-pro B cells gave rise to CD19^+^ B cells at high frequency, while PDCA-1^+^ pDCs in this fraction did not. Interestingly, the presence of PDCA-1^+^ pDCs within CLPs may help to explain the conflicting results regarding the origin of these cells.

## Introduction

The generation of B lineage lymphocytes from multipotent hematopoietic progenitors (MPP) is an ordered process orchestrated by genetic networks that initiate activation of the lymphoid lineage developmental program followed by specification and commitment to the B cell fate. At present, at least eight progenitor stages have been characterized between MPP and naïve B cells [1]–[4]. The various progenitor stages are distinguished by differential expression of combinations of cell surface markers, and regulated expression of genes that drive B cell development. However, it has become increasingly evident that some commonly used cell surface marker combinations do not adequately discriminate B cell precursors within transitional subsets from other lymphoid cells at various stages in their developmental programs. In some cases, this limitation has impeded the precise identification of developmental stage specific roles of regulatory factors in B lineage specification and commitment.

In particular, the developmental stages in which a common lymphoid progenitor (CLP) differentiates into a committed pro-B cell that has lost all other lineage potential is unclear. As B lineage precursors progress from CLPs to Pre-pro B cells to pro-B cells, the inability to purify B cell progenitors based on cell surface markers alone has led to the use of integrated reporters under the control of regulatory elements turned on during B cell differentiation including Rag1-GFP [5], [6] and λ5-hCD25 [7] to purify the earliest specified and committed B cell progenitors [8], [9]. The Rag1-GFP^+^ CLP population is heterogenous as well [8]. Single-cell PCR analysis revealed that while all the cells expressed EBF1, only half expressed either Pax5 or Pou2af1 [8]. Recently, Ly6D was discovered as a marker that could distinguish between cells with multi-lineage lymphoid potential and those specified to the B cell lineage within the CLP population [3]. Ly6D^−^ CLPs termed ALPs (all-lymphoid progenitors) give rise to T, B and NK cells, while Ly6D^+^ CLPs termed BLPs (B-cell biased lymphoid progenitors) give rise almost exclusively to B cells but very few T cells or NK cells in vivo [3]. While Ly6D can differentiate ALPs from BLPs within CLPs, BLPs are not a homogenous population. Differentiation of BLPs using in vitro culture resulted in the production of CD11c^+^ DCs in addition to CD19^+^ B cells [3]. Therefore, additional markers are needed to separate each stage of B cell progenitors within the CLP and Pre-pro B populations. Here, we demonstrate that PDCA-1^+^SiglecH^+^ plasmacytoid dendritic cells (pDCs) co-purify with BLPs and Pre-pro B cells. Once the pDC are removed using PDCA-1, the resulting PDCA-1^−^ BLPs and Pre-pro B cells populations express high levels of a Rag1-GFP reporter, indicating that these cells have initiated the B cell program. Once PDCA-1^+^ pDC are removed from the BLP and Pre-pro B populations, it revealed that the block in B cell development in Flt3-ligand and IL-7Rα knockout mice occurs at the ALP stage.

## Results

**Figure 1:**
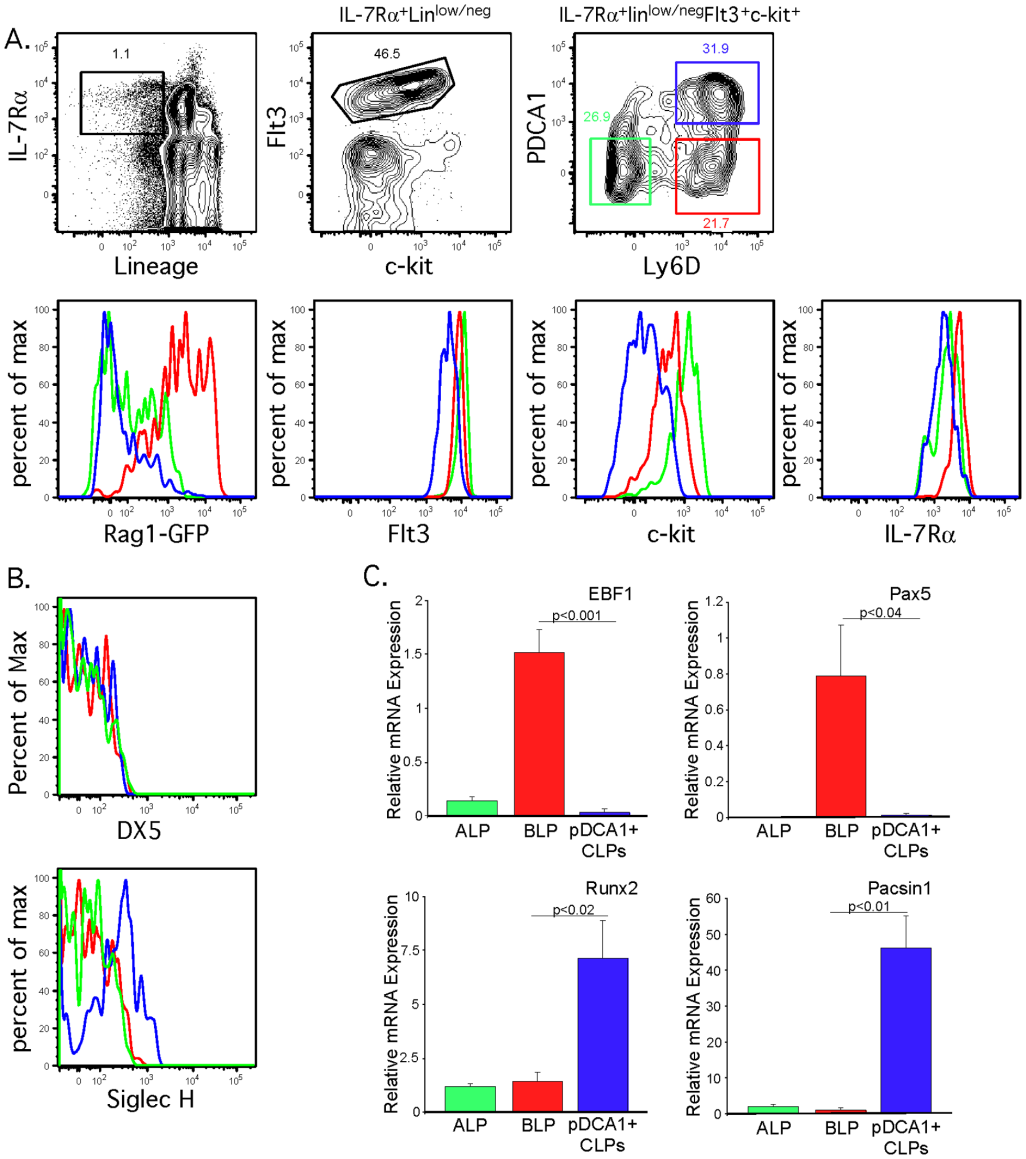
Identification of a plasmacytoid dendritic cell subset within the common lymphoid progenitor population. (A) Flow cytometry analysis was used to examine CLP populations from Rag1-GFP mice. Cells were first gated according to expression of IL-7Rα and low/negative of expression of lineage markers using a lineage cocktail containing antibodies against CD3ε, TCRβ, TCRγδ, B220, CD19, Ly6C, Ter119, CD11c, CD11b, Gr-1, NK1.1 and CD8α (left panel). These IL-7Rα^+^lineage^low^ cells were examined for expression of c-kit and Flt3 (middle panel). CLPs were identifed as IL-7Rα^+^lineage^low^ c-Kit^lo^Flt3^+^. CLPs were separated into three populations based on expression of Ly6D and PDCA-1 (right panel): ALP (Ly6D^−^PDCA-1^−^, green gate), BLP (Ly6D^+^PDCA-1^−^, red gate), and PDCA-1^+^ CLPs (Ly6D^+^PDCA-1^+^, blue gate). Each population was examined for expression of Rag1-GFP reporter, Flt3, c-kit and IL-7Rα. The analysis shown is representative of 4 mice analyzed in 3 independent experiments. (B) Analysis of expression of Siglec H and DX5 in ALP (green gate), BLP (red gate) or PDCA-1^+^ CLPs (blue gate) populations, gated as in figure 1(A). (C) Quantitative PCR was performed on ALPs, BLPs and PDCA-1^+^ CLPs (color coded as in the gating shown in figure 1(A)). Each population was independently isolated by FACS sorting using the gating strategy in (A). Relative expression of EBF1, Pax5, Runx2 and Pacsin1 is shown. Data shown is the average of 4 independently sorted samples from 4 WT mice, and error bars reflect the standard error of the mean. Data was normalized to the expression of each gene in one of the four WT BLP samples. Significance was quantified using unpaired two-tailed t test (GraphPad Prism).

Plasmacytoid dendritic cells (pDCs) share many cell surface markers with B lymphoid progenitors, and have traditionally been excluded from lineage cocktails using Ly6C and/or CD11c [3], [10]. However, as dendritic cells are very heterogenous, with distinct populations expressing low levels of CD11c [11] or Ly6C [12], it was possible that these two markers alone would be insufficient for exclusion of dendritic cells in the lineage cocktail used to examine lymphoid progenitors. In particular, plasmacytoid dendritic cells (pDCs) express high levels of Ly6D, the marker used to define BLPs within the CLP gate [3]. PDCA-1 is found on the surface of pDCs [13], and was used to examine whether pDCs remained after lineage exclusion for examination of CLPs. Our gating strategy for CLPs is shown in figure 1A, gating first on lin^low^ (lineage cocktail containing antibodies against CD3ε, TCRβ, TCRγδ, B220, CD19, Ly6C, Ter119, CD11c, CD11b, Gr-1, NK1.1 and CD8α), IL-7Rα^+^, c-Kit^low^ and Flt3^high^ as was performed in [3]. This CLP population was then examined for Ly6D expression to differentiate ALPs from BLPs [3], and for PDCA-1 expression. PDCA-1 positive cells comprised a substantial proportion of Ly6D^+^ BLPs, but were not found in the earlier Ly6D^−^ ALPs. The CLP pool is heterogenous, and transgenic reporters such as Rag1-GFP and λ5 are often used to identify subpopulations destined to become B cells [8]. We hypothesized that the heterogeneity of Rag1-GFP reporter expression within the BLP population is due to contaminating PDCA-1^+^ cells. As shown in figure 1A, PDCA-1^−^Ly6D^+^ BLPs expressed high levels of the Rag1-GFP reporter while few PDCA-1^+^ CLPs were Rag1-GFP positive (even lower than in Ly6D^−^ ALPs). We examined expression of Flt3, c-kit and IL-7Rα in each of these CLP populations, however due to considerable overlap between the histograms, these markers cannot be used to distinguish PDCA-1^+^ CLPs from PDCA-1^−^ BLPs. Therefore, the lack of PDCA-1 expression can be used to distinguish a subpopulation of Ly6D^+^ BLPs that express high levels of Rag1-GFP reporter and are specified but not yet committed to the B cell lineage [3].

To confirm the identity of these PDCA-1^+^ CLPs, we examined additional cell surface markers as well as expression of genes characteristic of the B cell and pDC lineages by Q-PCR. The PDCA-1^+^ CLPs expressed Siglec H, which is primarily expressed on pDCs and not on other hematopoietic lineages [14], and did not express DX5 (figure 1B). To confirm that these PDCA-1+ CLPs were pDCs, we examined the expression of Runx2 [15] and Pacsin1 [16]. Previous analysis by the Immgen Consortium [17], demonstrated that Pacsin1 and Runx2 are expressed by pDCs but not any conventional DC lineage. In addition, analysis of the Immgen database showed that Pacsin1 is not expressed by any B cell lineage and that Runx2 is expressed in B cell progenitors albeit at a considerably lower level than pDCs. Thus, differential expression of Runx2 and Pacsin1, in comparison to the B lineage genes EBF1 [18] and Pax5 [18] which are required for B cell specification and commitment, could differentiate B lineage progenitors from pDCs. As shown in figure 1B, expression of these genes was low in ALPs, and only EBF1 and Pax5 (but not Runx2 or Pacsin1) were upregulated in BLPs. The PDCA-1^+^ CLPs expressed high levels of Runx2 and Pacsin1, but not EBF1 or Pax5, indicating that they were pDCs. Although CD11c and Ly6C were included in the lineage cocktail, pDC populations have been identified with low levels of both CD11c [11] or lack of Ly6C [12], indicating that these markers may not be sufficient to eliminate pDCs in a lineage cocktail. Here, we show that PDCA-1 is an effective marker to discriminate pDCs from BLPs within the CLP population.

**Figure 2:**
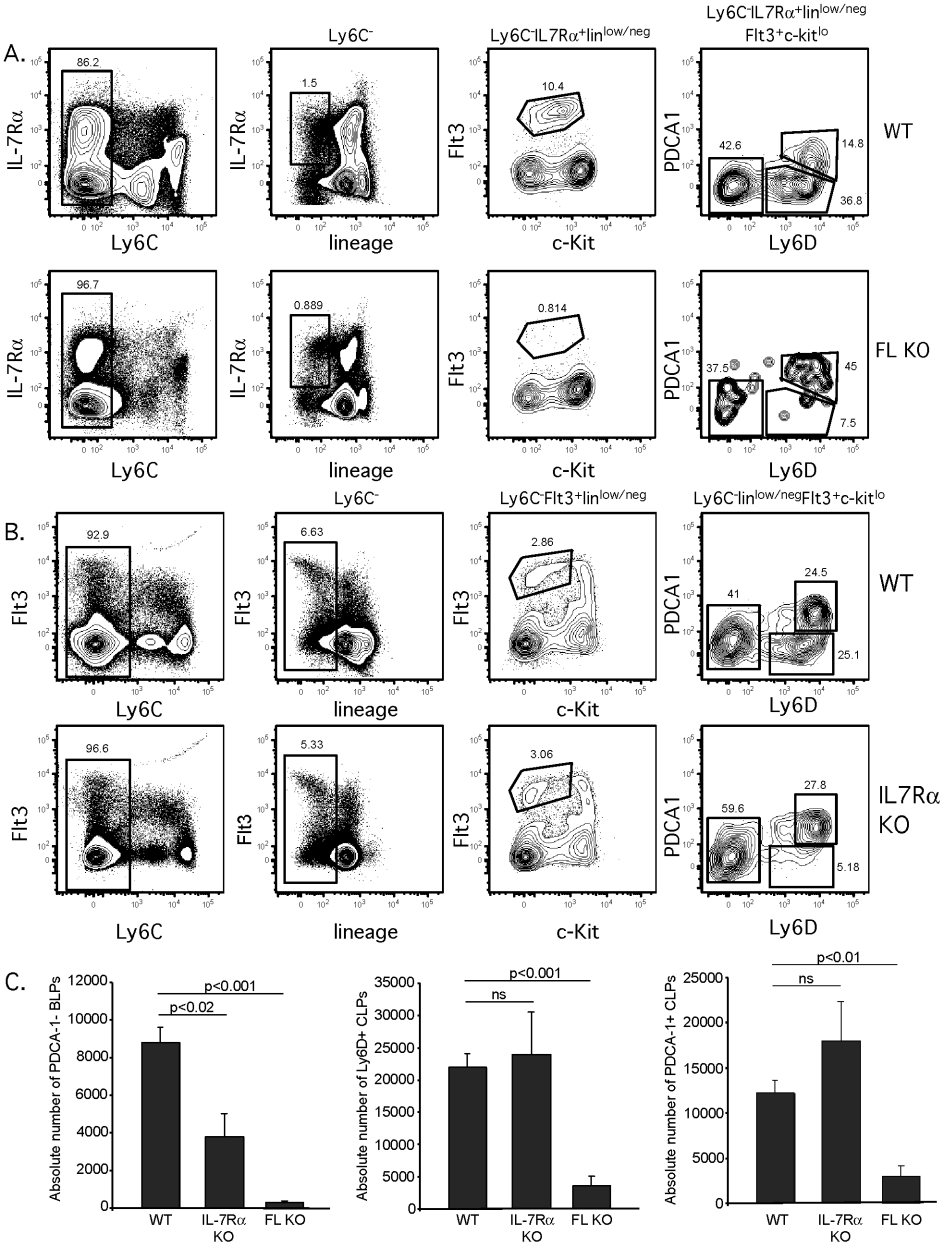
Severe decrease in BLPs in IL-7Rα and Flt3-ligand deficient mice revealed upon exclusion of pDCs. (A) WT and Flt3-ligand (FL) knockout (KO) mice were examined for ALPs and BLPs using the gating strategy described in Figure 1A, except that Ly6C was analyzed separately from lineage (using a lineage cocktail as in Figure 1A except lacking Ly6C). Data shown is representative of 4 WT and 4 FL KO mice from 4 independent experiments. (B) WT and IL-7Rα KO mice were examined for ALPs and BLPs using the gating strategy as in Figure 2A, except that IL-7Rα expression was not used to define CLPs. Data shown is representative of 4 WT and 4 FL KO mice from 4 independent experiments. (C) The absolute number of PDCA-1^−^ BLPs, total Ly6D^+^ CLPs and PDCA-1^+^ pDCs within the CLP population per femur was calculated in WT, FL KO and IL-7Rα KO mice. In the left panel, PDCA-1^−^ BLPs were analyzed after exclusion of PDCA-1^+^ pDCs from the Ly-6D^+^ CLP gate. In the middle panel, total Ly6D^+^ CLPs were analyzed without exclusion of PDCA-1^+^ pDCs from the Ly6D^+^ CLPs gate. In the right panel, total PDCA-1^+^ pDCs within the CLP population were examined. Statistical analysis was performed using an unpaired two-tailed t test (GraphPad Prism).

The presence of PDCA-1^+^ pDCs in the the Ly6D^+^ CLP population could mask a defect in BLPs in genetically modified mice. In particular, there are conflicting results when the block in B cell development occurs in IL-7Rα knockout (KO) mice. Previous work by others identified the block at the CLP stage [19], the Pre-pro B stage [20], or the Pro-B (Fraction B) cell stage [21], [22]. Due to the overlap in cell surface markers between CLPs and PDCA-1+ SiglecH+ pDCs (figure 1), the presence of pDCs could be misinterpreted as CLPs. Therefore, we included PDCA-1 in our analysis of the BLP population in IL-7Rα KO mice. We also included Ly6C separately from the lineage markers to confirm that the PDCA-1^+^ CLPs were Ly6C low/negative. This analysis demonstrates that the majority of Ly6D^+^ CLPs in IL-7Rα KO (Figure 2B) are PDCA-1^+^ pDCs and not BLPs, indicating that the block in lymphocyte development in IL-7Rα KO is at the transition between ALPs and BLPs. Similar results were obtained in analysis of Flt3-ligand (FL) KO mice that are impaired in their ability to generate B cell progenitors [23], [24]. We also analyzed absolute numbers of (PDCA-1^−^) BLPs as well as total Ly6D^+^ CLPs (which includes both PDCA-1^−^ BLPs and PDCA-1^+^ pDCs). Analysis of total Ly6D^+^ CLPs revealed no differences between WT and IL-7Rα KO mice. However, when PDCA-1^+^ pDCs were excluded, the number of BLPs was significantly lower in IL-7Rα KO mice (Figure 2C). Thus, removal of PDCA-1^+^ pDC from the BLP gate revealed a defect in B cell development in IL-7Rα KO mice and FL KO mice at the ALP stage.

**Figure 3:**
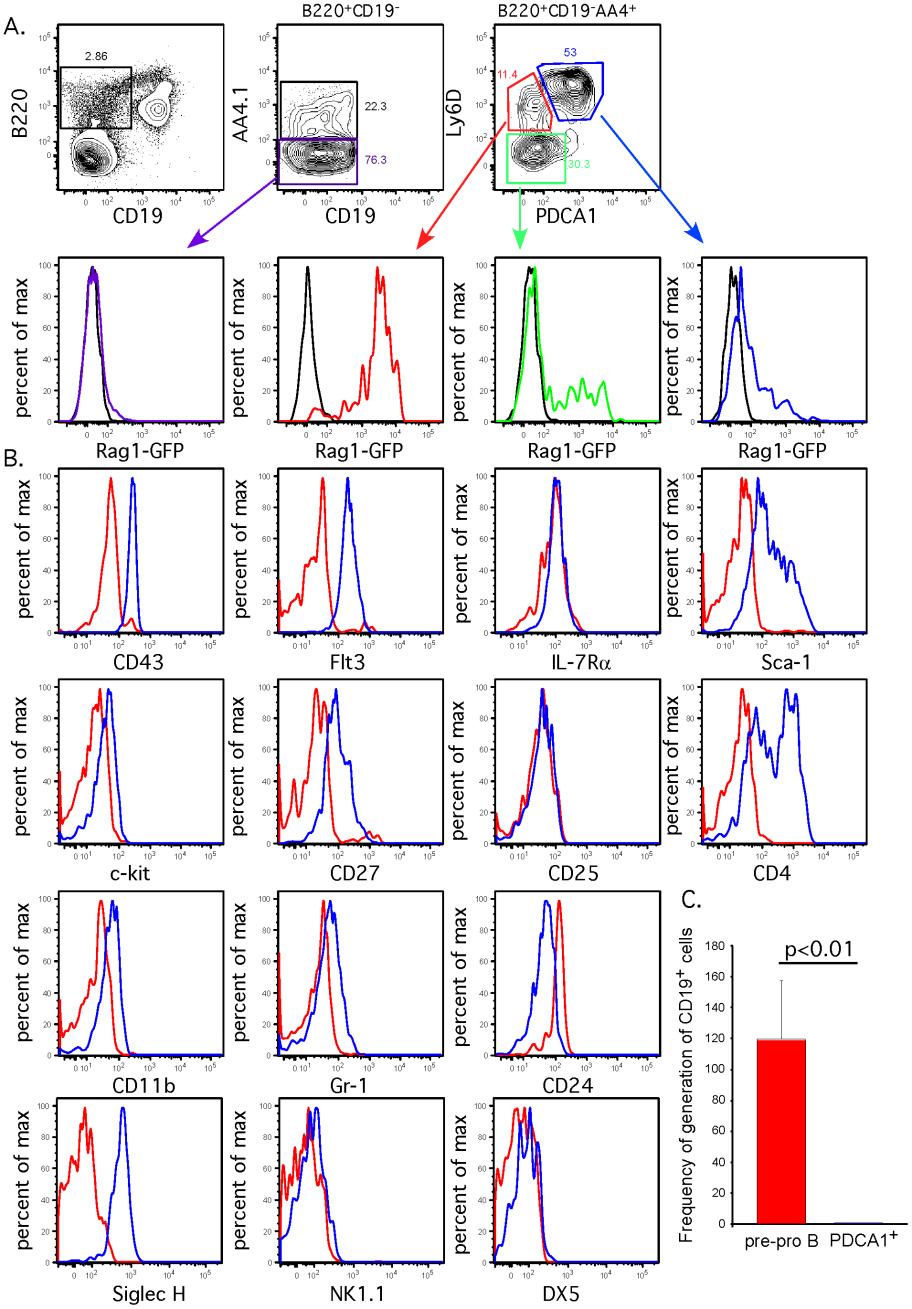
pDCs also contaminate the Pre-pro B population. (A) Flow cytometry analysis was performed on bone marrow cells to subfractionate B220^+^CD19^−^ Fraction A. Using AA4.1, Ly6D and PDCA-1, four populations were distinguished within the B220^+^CD19^−^ population. Each of the gated populations defined were examined for expression of Rag1-GFP mice. The black histogram is the negative control, showing lack of GFP expression in WT mice, with each population color coded to the gating of each population highlighted in A (Purple is B220^+^CD19^−^AA4.1^−^; Red is B220^+^CD19^−^AA4.1^+^Ly6D^+^PDCA-1^−^; Blue is B220^+^CD19^−^AA4.1^+^Ly6D^+^PDCA-1^+^; and Green is B220^+^CD19^−^AA4.1^+^Ly6D^−^PDCA-1^+^). Shown are representative plots of 4 mice from 3 independent experiments. (B) Examination of cell surface markers to distinguish the two populations of B220^+^CD19^−^AA4.1^+^Ly6D^+^ cells. Histograms are color coded as in A and B, with red representing expression in B220^+^CD19^−^AA4.1^+^Ly6D^+^PDCA-1^−^ cells and blue representing expression in B220^+^CD19^−^AA4.1^+^Ly6D^+^PDCA-1^+^ cells. Shown are representative plots from four mice analyzed in two independent experiments. (C) B lineage potential of B220^+^CD19^−^AA4.1^+^Ly6D^+^PDCA-1^−^ cells (red gate in figure 3A) and B220^+^CD19^−^AA4.1^+^Ly6D^+^PDCA-1^+^ cells (blue gate in figure 3A) was examined by sorting each population onto OP9 stromal cells in media containing IL-7, Stem cell factor (SCF) and Flt3 ligand. After 4 days, cells were stained with CD19 and the total number of CD19^+^ cells generated compared to the number of input sorted cells per well was calculated (frequency of generation of CD19^+^ cells). The frequency was calcuated from 7 independent wells per sample from 3 independent sorts. Statistical analysis was performed using an unpaired two-tailed t test (GraphPad Prism).

Similarly, pDCs are also found within the heterogenous B220^+^CD19^−^ Pre-pro B population [25]. Pre-pro B cells have been shown to express both AA4.1/CD93 [26], [27] and Ly6D [3]. As shown in figure 3A, there were four major populations within the B220^+^CD19^−^ gate. Of the cells that express both AA4.1 and Ly6D, a substantial percentage were PDCA-1^+^ (blue gate), and a smaller percentage were negative and thus potentially represented Pre-pro B cells (red gate), the immediate precursor to committed CD19^+^ B cell progenitors. As Rag1 gene expression precedes commitment to the B cell lineage [6], expression of a Rag1-GFP transgenic reporter [5] was examined in each of the four populations in the B220^+^CD19^−^ gate. High levels of Rag1-GFP expression was only found within the AA4.1^+^Ly6D^+^PDCA-1^−^ population, consistent with their identification as Pre-pro B cells (Figure 3B), but not in the AA4.1^+^Ly6D^+^PDCA-1^+^ population. To further characterize the AA4.1^+^Ly6D^+^PDCA-1^−^ and AA4.1^+^Ly6D^+^PDCA-1^+^ populations, additional markers were examined (Figure 3C). Pre-pro B cells (red line in histograms) expressed intermediate levels of CD43, and were Flt3^low^IL7Rα^+^Sca1^−^c-kit^−^CD27^low^CD25^−^CD4^−^CD11b^−^Gr-1^−^. In contrast, the B220^+^CD19^−^AA4.1^+^Ly6D^+^ population that also expressed PDCA1 (blue line in histogram) were Flt3^high^IL7Rα^+^Sca1^+^c-kit^low^CD27^+^CD25^−^CD4^+^CD11b^low^Gr-1^+^CD43^hi^. The expression of CD4, Gr-1 and low level of CD11b expression is consistent with this population being pDCs [11], [28]. In addition, the PDCA-1^+^ cells in the B220^+^CD19^−^ population also expressed Siglec-H, but not DX5 or NK1.1, demonstrating their identity as pDCs. We analyzed the ability of the AA4.1^+^Ly6D^+^PDCA-1^−^ cells (red gate) and AA4.1^+^Ly6D^+^PDCA-1^+^ cells (blue gate) to generate B cells *in vitro*. Each population was sorted as in Figure 3A, and cultured on OP9 stromal cells with exogenous IL-7, Flt3-ligand and SCF. After 4 days, the cultures were analyzed by flow cytometry and the number of CD19^+^ cells produced compared to the number of progenitors plated were compared. As shown in figure 3C, the AA4.1^+^Ly6D^+^PDCA-1^−^ cells gave rise to a high frequency of CD19^+^ cells, while almost none were produced from the AA4.1^+^Ly6D^+^PDCA-1^+^ population. Therefore, the majority B220^+^CD19^−^AA4.1^+^Ly6D^+^ cells are PDCA-1^+^ pDCs without B progenitor potential and a smaller fraction are (PDCA-1^−^) Pre-pro B cells. This population of Pre-pro B cells, isolated after exclusion of PDCA-1+ pDCs, expresses high levels of the Rag1-GFP reporter. Overall, the frequency of AA4.1^+^Ly6D^+^PDCA-1^−^ Pre-pro B cells within the B220^+^CD19^−^ gate is approximately 1–2%.

**Figure 4:**
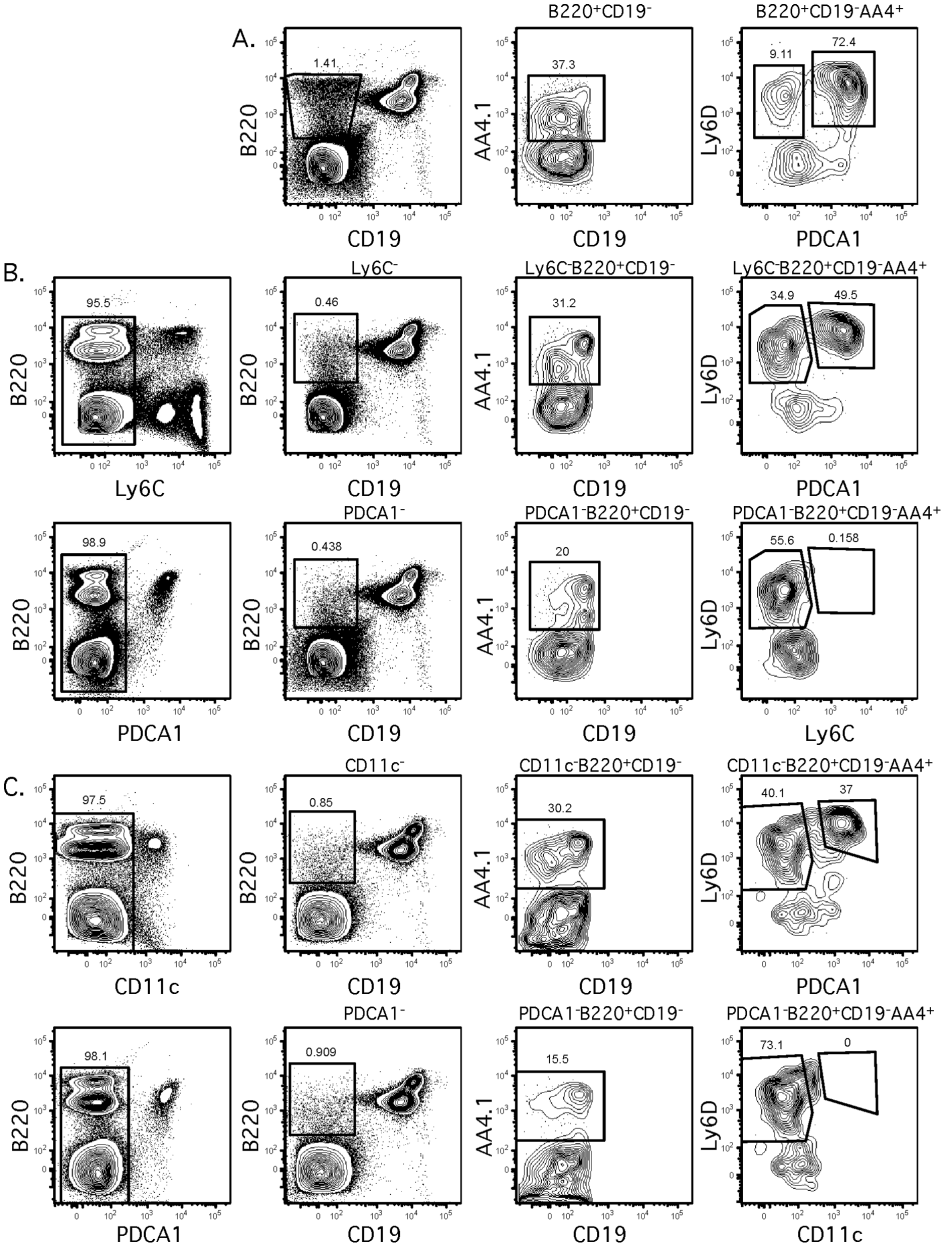
Neither Ly-6C nor CD11c eliminates PDCA-1+ pDCs from Pre-pro B cells. (A) Cells within the Pre-pro B gate were analyzed as in Figure 3A. (B) Cells within the Pre-pro B gate were analyzed as in Figure 3A, except that Ly-6C was also included. In the upper panels, cells were first gated to exclude Ly-6C. Exclusion of Ly-6C+ cells did not remove PDCA-1+ cells from Pre-pro B cells. In the lower panel, cells were first gated to exclude PDCA-1+. Exclusion of PDCA-1+ cells removed all Ly-6C+ cells from Pre-pro B cells. (C) Cells within the Pre-pro B fraction were analyzed as in Figure 3A, except that CD11c was also included. In the upper panels, cells were first gated to exclude CD11c. Exclusion of CD11c+ cells did not remove PDCA-1+ cells from Pre-pro B cells. In the lower panel, cells were first gated to exclude PDCA-1+. Exclusion of PDCA-1+ cells removed all CD11c+ cells from Pre-pro B cells.

The use of PDCA-1 clearly distinguishes Rag1-GFP^hi^ Pre-pro B cells from pDCs within the AA4.1^+^Ly6D^+^ Pre-pro B population. Previously, others groups have used either Ly6C or CD11c to exclude dendritic cells from Pre-pro B cells [29], . However, based on our work in CLPs above, neither Ly6C nor CD11c may be sufficient to remove pDCs from Pre-pro B cells. This was analyzed in figure 4, to determine whether first gating for Ly6C^−^ cells (figure 4B) or CD11c^−^ cells (figure 4C) could eliminate PDCA-1^+^ pDCs from AA4.1^+^Ly6D^+^ Pre-pro B cells (figure 4A). Neither Ly6C nor CD11c was sufficient to remove PDCA-1^+^ pDCs from AA4.1^+^Ly6D^+^ Pre-pro B cells. However, if PDCA-1^+^ were removed first, then no Ly6C or CD11c expressing cells were found in the AA4.1^+^Ly6D^+^ fraction. This demonstrates that PDCA-1 is required to remove pDCs from AA4.1^+^Ly6D^+^ Pre-pro B cells.

**Figure 5:**
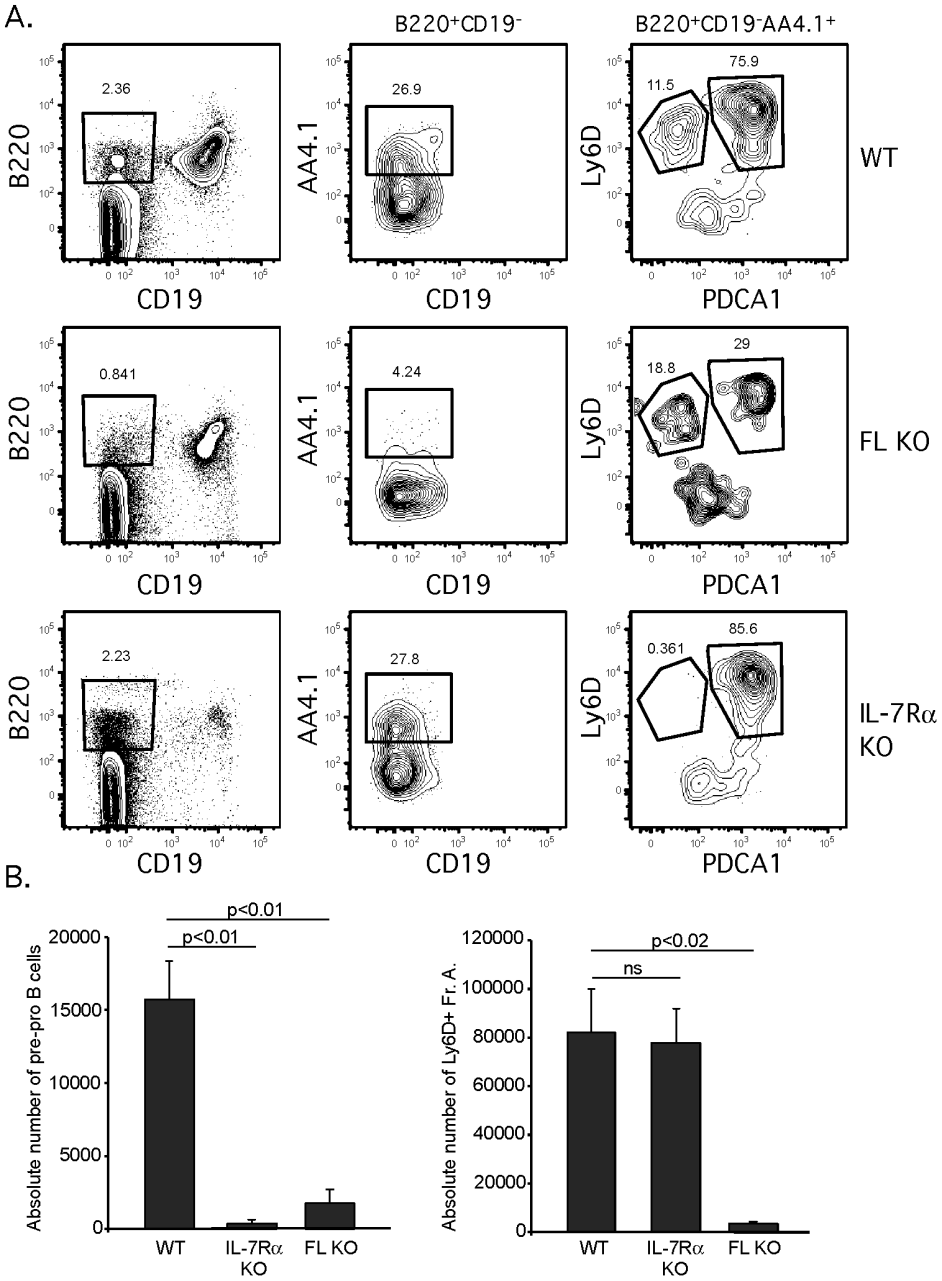
Severe decrease in Pre-pro B cells in IL-7Rα and Flt3-ligand deficient mice revealed upon exclusion of pDCs. A. WT, IL-7Rα knockout, and Flt3 ligand knockout mice were analyzed for the presence of Pre-pro B cells as described in Figure 3. Data shown is from a representative experiment that was replicated independently four times. B. Absolute numbers of Pre-pro B cells from WT, IL-7Rα knockout, and Flt3 ligand knockout mice. On the left is the analysis of Pre-pro B cells (B220^+^CD19^−^AA4.1^+^Ly6D^+^) after elimination of PDCA-1+ pDCs. On the right is the analysis of Pre-pro B cells without elimination of PDCA-1+ pDCs. Error bars represent SEM from 4 mice in each group analyzed in 4 independent experiments. Statistical analysis was performed using an unpaired two-tailed t test (GraphPad Prism).

Next, we utilized this new scheme to analyze Pre-pro B cells in IL-7Rα KO mice [21] and Flt3 ligand KO mice [24]. These mice share deficiencies in CLPs and B cell precursors, but have been reported to exhibit normal numbers of Pre-pro B cells [31], [32]. However, as PDCA-1^+^SiglecH^+^ pDCs contaminate the Pre-pro B population, a defect in Pre-pro B cells may have been masked by pDCs. As shown in Figure 5, severe reductions in Pre-pro B cells, both in frequency and absolute numbers, were found in mice deficient for IL-7Rα and Flt3 ligand, using our modified gating scheme excluding PDCA-1^+^ pDCs, consistent with expectations based on defects in the CLP and subsequent pro-B cell populations. In particular, we compared the absolute number of Pre-pro B cells in these mice, either using AA4.1^+^Ly6D^+^ to define Pre-pro B cells (right panel) or after exclusion of PDCA-1^+^ pDCs (left panel). Without exclusion of PDCA-1^+^ pDCs, the number of Pre-pro B cells in IL-7Rα-deficient mice was similar to WT. However, after exclusion of PDCA-1^+^ pDCs, a nearly complete deficiency in Pre-pro B cells in IL-7Rα-deficient mice was revealed. Therefore, PDCA-1 is an important marker to exclude pDCs from both the Pre-pro B and BLP gates.

## Discussion

Here, we demonstrate that PDCA-1^+^SiglecH^+^ plasmacytoid dendritic cells (pDCs) co-purify with BLPs and Pre-pro B cells. Removal of PDCA-1^+^ pDCs separates B cell progenitors that express high levels of Rag1-GFP reporter from Rag1-GFP^low/neg^ pDCs within the BLP and Pre-pro B populations. In general, there are many similarities in expression of cell surface markers between pDC and B cell progenitors. They both express IL-7Rα, Flt3, c-kit, as well as markers traditionally identified as belonging exclusively to the B cell lineage including AA4.1, B220 and Ly6D. Thus, it is perhaps not surprising that pDCs copurify with CLPs and Pre-pro B cells. Given the similarity in cell surface markers, B cell progenitors may also copurify with pDCs. Therefore, PDCA-1 should be used in conjunction with Ly6C and CD11c to remove pDCs from B cell progenitor analysis. The presence of pDCs within the BLP and AA4.1^+^Ly6D^+^ Pre-pro B populations would confound analysis of differential gene expression between WT and genetically altered mice if these cells are not accounted for in the cell purification scheme, as they also express several genes characteristically associated with B cell lineage commitment and specification including Pax5, EBF and CD79a/mb1 [11]. For example, we demonstrate that BLPs and Pre-pro B cells are absent in IL-7Rα knockout mice, but PDCA-1^+^AA4.1^+^Ly6D^+^ cells in the Pre-pro B gate and PDCA-1^+^ pDCs in the CLP gate are present. Therefore, with prior gating strategies, PDCA-1^+^ pDCs would be mistaken for B cell progenitors cells in these mice, and would express a genetic signature that contained elements usually associated with the B cell lineage. By gating out PDCA-1^+^ pDCs, we demonstrate that the block in B cell development in IL-7Rα KO and FL KO mice is at the ALP stage.

Recently, two groups used different 12 parameter flow cytometry profiles to identify Pre-pro B cells [3], [4], however neither groups specifically excluded pDCs. Here, we show that B220^+^CD19^−^ B cell precursors enriched for Pre-pro B cells can be divided into 4 populations using the markers AA4.1, Ly6D and PDCA-1, only one of which (AA4.1^+^Ly6D^+^PDCA-1^−^) has uniformly high levels of Rag1-GFP reporter expression. Thus, Rag1-GFP^+^ Pre-pro B cells can be identified using only 5 markers. In our analysis, Pre-pro B cells express intermediate levels of CD43, consistent with previous results (Figure 1D, [4]). Often, the B220^+^CD43^high^ population is fractionated into CD19^−^ pre-pro B and CD19^+^ pro-B cell populations [33], [34]. However, our analyses show that these B220^+^CD43^high^CD19^−^ cells are PDCA-1^+^ pDCs and not Pre-pro B cells.

The discovery of pDCs within the BLP population may explain the conflicting data with regards to their origin [35]. pDCs have been shown to arise from lymphoid-biased multipotent progenitors (LMPPs), common myeloid progenitors (CMPs), as well as CLPs [36]–[39]. However, our data demonstrates that PDCA-1^+^ pDCs persist in the CLP gate, even after utilizing a lineage cocktail that contains Ly6C, CD11c, CD11b and Gr-1. Without the use of PDCA-1, pDCs co-purify with CLPs and could therefore be expanded upon in vitro culture of CLPs, and thus confuse the analysis and interpretation regarding the origin of pDCs.

Here, we also show there is heterogeneity in the pDC population as well. We identified PDCA-1^+^SiglecH^+^ pDC which are not removed from the lineage low population using Ly6C and CD11c that co-purify with CLPs and Pre-pro B cells. Where these pDCs fall within the continuum of pDC development is unclear. Onai et al [36] recently found a unique progenitor population (Lin-c-kit^int/lo^Flt3^+^M-CSFR^−^) that produce pDC at high frequency, but lack expression of PDCA-1 or Siglec H. Differentiation of these pDC precursors in culture produces cells with high levels of PDCA-1 and Siglec H expression [36]. The pDCs that copurify with CLP and Pre-pro B cells express both PDCA-1 and Siglec H, and therefore would be downstream of these newly identified pDC precursors. Whether these cells represent a developmental intermediate during pDC development and differentiation, or a unique lineage, is currently under investigation.

## Materials and Methods

### Ethics statement

This study was carried out in strict accordance with the recommendations in the Guide for the Care and Use of Laboratory Animals of the National Institutes of Health. The work was performed with the approval of the Mayo Clinic Institutional Animal Care and Use Committee (OLAW Assurance number A3291-01).

### Mice

Flt3 ligand knockout mice [24] were received from Taconic Farms and IL-7Rα knockout mice [21] were received from Jackson Laboratories. Rag1-GFP mice were generated and provided by Dr. Nobuo Sakaguchi [5]. WT mice used were C57BL/6 from either Taconic Farms or Jackson Laboratories. All animals were analyzed between 8 and 20 weeks of age.

### Flow cytometry

Antibodies were from eBioscience, Biolegend or Becton Dickinson. All samples were gated on size, and doublet excluded during analysis using FSC-H/FSC-W as well as SSC-H/SSC-W. The lineage depletion cocktail for analysis of CLPs contained twelve antibodies to exclude differentiated hematopoietic populations: CD3ε, TCRβ, TCRγδ, B220, CD19, Ly6C, Ter119, CD11c, CD11b, Gr-1, NK1.1 and CD8α, all conjugated to APC. Other antibodies used in analysis of CLPs included Flt3-PE, IL-7Rα-biotin/streptaviding PE-Cy7, c-kit-APC-Cy7, and PDCA-1 efluor450. For experiments analyzing CLPs with Ly6C, an APC lineage cocktail as above without Ly6C was used and Ly6C-PerCP-Cy5.5 was used. For pre-pro B cells in wild-type mice, 10 million bone marrow cells were stained with the following antibodies: Ly6D-FITC (BD Biosciences); CD93/AA4.1-PE (eBioscience); PDCA-1 PerCP-efluor 710 (eBioscience); CD19-PECy7 (eBioscience) and B220-APC-Cy7 (Biolegend). For experiments in Pre-pro B cells analyzing Rag1-GFP expression, Ly6D-efluor450 was used. Analysis was performed on a Canto or LSRII flow cytometer (Becton Dickinson), and analyzed using FlowJo software (TreeStar), with 0.5–1.0 million events collected per sample. In all experiments except where efluor450 was used, DAPI was used to exclude dead cells.

### Quantitative PCR

ALPs, BLPs and PDCA1+ CLPs from 4 WT mice (using the gating strategy described in Figure 1) were sorted on an Aria flow cytometer (Becton Dickinson). RNA was generated using a RNeasy micro kit (Qiagen), and amplified cDNA generated using Ovation PicoSL WTA kit v2 (NuGen), both as per manufacturer's instructions. Taqman probes (Applied Biosystems) for EBF1, Pax5, Runx2, Pacsin1 and HPRT were used. An ABI StepOne Plus System (Applied Biosystems) was used and expression relative to HPRT was calculated with the 2-ΔΔCT method [40].

### B cell differentiation cultures

Single cell bone marrow suspensions were generated from WT mice. B lineage precursors wre enriched by magnetic depletion of Ter119^+^, Mac1^+^, CD3ε^+^, Gr-1^+^ and IgM^+^ cells. B lineage enriched bone marrow cells were stained with antibodies to B220, CD19, AA4.1, Ly6D and PDCA-1. B220^+^CD19^−^AA4.1^+^Ly6D^+^PDCA-1^−^ cells (red gate, as shown in Figure 3A) and B220^+^CD19^−^AA4.1^+^Ly6D^+^PDCA-1^+^ cells (blue gate, as shown in Figure 3A) were sorted using a FACS-Aria (BD Biosciences). The sorted cells were plated on OP9 stromal cells in IMDM media with 10% FBS, L-Glutamine, β-mercaptoethanol, 10 ng/ml human IL-7, 10 ng/ml Stem Cell Factor and 10 ng/ml human Flt3-ligand. All cytokines were from Peprotech. After 4 days in culture, the cells were harvested, and analyzed for expression of CD19.
